# Sustainability in Quality Improvement: Outcomes of a Novel Pediatric Virtual Urgent Care in Ontario, Canada

**DOI:** 10.5811/westjem.54076

**Published:** 2026-07-28

**Authors:** Emma Lim, Julie Strychowsky, Anna C Gunz, Sheena Belisle

**Affiliations:** * McMaster University, Michael G DeGroote School of Medicine, Hamilton, Ontario, Canada; † Western University, Schulich School of Medicine and Dentistry, Department of Surgery, London, Ontario, Canada; ‡ London Health Sciences Centre, Department of Otolaryngology - Head and Neck Surgery, London, Ontario, Canada; § Western University, Schulich School of Medicine and Dentistry, Department of Paediatrics, London, Ontario, Canada; || Child Health Research Institute, London, Ontario, Canada

## Abstract

**Introduction:**

Virtual care is expected to play an increasingly important role in the Canadian healthcare system, with the 2023–2024 Health Canada Departmental Plan outlining the advancement of digital health solutions as a focal point. However, key outcomes of transitioning from traditional in-person healthcare appointments to a virtual model of care are not yet well-described. Here, we apply the Sustainability in Quality Improvement (SusQI) framework to patients seen in a novel pediatric virtual urgent care (VUC) program to quantify the environmental, economic, and social impacts of virtual care.

**Methods:**

We conducted a retrospective analysis of provincial pediatric VUC visits staffed by pediatric emergency physicians and nurse practitioners during the 2020–2025 fiscal years. We focused on three key metrics: (1) economic value, measured as travel costs avoided; (2) environmental value, measured in fuel saved (L) and carbon emissions reduced (kg CO_2_ eq); and (3) social value, assessed using Ontario Marginalization (ON-Marg) Index quintiles.

**Results:**

A total of 12,900 virtual visits were completed during the study period, 99.9% via the Webex platform. Net savings totaled $998,100. Environmental benefits included 128,700 L of fuel saved and 384,200 kg CO_2_ eq emissions avoided. Summary ON-Marg Index scores were similar between groups (2.87 for in-person emergency department (ED) visits; 2.91 for virtual visits). Data collected between July 2023–April 2025 show that 10.6% (353) of visits resulted in subsequent in-person ED presentation.

**Conclusion:**

Virtual urgent care resulted in significant financial and environmental savings per visit. These benefits included reduced financial and time burdens for families, lower hospital-related expenditures, improved environmental performance, and diversion of low-acuity cases from the ED. Comparable ON-Marg scores between patients seen virtually and in-person suggest accessibility across socioeconomic groups. These findings indicate that pediatric VUC is a viable complement to traditional care, providing meaningful cost and environmental savings while ensuring equitable access.

## INTRODUCTION

Climate change is an area of significant concern within healthcare. The effects of climate change pose an imminent threat to human health, increasing morbidity and mortality, widening existing social disparities, and increasing the burden on healthcare services.^1^ The development of sustainable healthcare systems can be achieved through mitigation policies aimed at reducing carbon emissions and by identifying ways to create more robust healthcare infrastructure.^2^ Virtual care is one promising avenue for carbon reduction, as it eliminates the need for patients to travel to clinics, reducing their reliance on fossil fuels.^3^,^4^ Virtual care may also improve access by reducing the time, distance, and effort required by patients attending appointments.^5^,^6^ Understanding the profile of carbon emissions associated with virtual and in-person care can guide the development of a more sustainable healthcare system.^7^ In 2020, as part of the COVID-19 pandemic response, the Ontario Ministry of Health approved $4 million in funding for regionally coordinated virtual urgent care (VUC) initiatives across Ontario.^8^ A 2023 cross-sectional survey evaluating patients using VUC services in Ontario found that 94% of patients rated their experience as 8/10 or greater, with more than 80% saying they felt confident managing their presenting concern.^9^ Virtual care continues to be used across multiple medical specialties. In 2021, during the pandemic, approximately 40% of all healthcare visits in Canada were virtual.^10^ The 2023–2024 Health Canada Departmental Plan outlined the advancement of digital health solutions as a focal point, indicating the increasingly important role virtual care is expected to play in the Canadian healthcare system.^11^

The primary goal of this paper was to examine the value of VUC in patients who would otherwise present to a pediatric emergency department (ED) by quantifying associated savings. A secondary goal was to explore the various impacts of its continued use as a substitute for in-person emergency medicine in applicable patients. We also aimed to evaluate disparities in access to care by comparing the demographic makeup of patients accessing virtual versus in-person emergency services. This assessment focused on three key areas: financial; environmental; and social considerations. This analysis was guided by the Sustainability in Quality Improvement (SusQI) framework, developed by the Centre for Sustainable Healthcare (CSH) in the United Kingdom.^12^ The SusQI framework focuses on the CSH principles of sustainable clinical practice and measures the health outcomes of a service against the Triple Bottom Line model introduced in 1997 by economist John Elkington. This model examines sustainable development through a multifaceted lens of social, environmental, and economic sustainability.^13^ Here, we used the SusQI framework to provide an understanding of the value of virtual care and the potential for expansion and application within other healthcare domains.

**Population Health Research Capsule**

What do we already know about this issue?

*Virtual care is expected to play an increasing role in healthcare, but its broader environmental, economic, and social impacts remain poorly characterized*.

What was the research question?

*To quantify the financial, environmental, and social impacts of pediatric virtual urgent care using the Sustainability in Quality Improvement framework*.

What was the major finding of the study?

*Virtual urgent care saved $998,100, 128,700 L of fuel and 384,200 kg CO_2_ across 12,900 visits with 10.6% directed to the emergency department*.

How does this improve population health?

*Virtual urgent care reduces emissions and financial burden while improving access to care, which supports the development of more sustainable and resilient healthcare systems*.

## METHODS

### Study Design

We conducted a retrospective analysis of patients presenting to the pediatric Ontario VUC program operating out of the hospital. This was part of a joint pilot initiative to offer virtual services to residents in southwestern Ontario. The service was publicly available and promoted through institutional communications and news articles. Individuals accessed the VUC through the Urgent Care Ontario website. Virtual visits were conducted using the Webex application, a secure videoconferencing platform. Pediatric patients were defined as individuals ≤ 17 years of age at the time of their visit. No exclusion criteria were applied. Data were sourced from the hospital virtual care dashboard, developed by the hospital decision support team. The dashboard collects data on all patients who received outpatient care at the hospital since 2020. All patients who used pediatric VUC during the fiscal years of April 2020–March 2025 were captured in our analysis.

Clinical variables were extracted automatically; therefore, no manual chart review was deemed necessary. Available variables include the volume of in-person and virtual visits, financial and travel savings associated with virtual visits, and Ontario Marginalization (ON-Marg) Index scores. We also collected ON-Marg scores for in-person ED visits occurring during the same time periods. Data on subsequent presentation to ED was captured by standardized fields within the clinical virtual care note indicating clinician recommendation to seek further care at an ED. In addition, we invited all patients who used pediatric VUC to complete a feedback survey one month after their visit which included questions regarding serious adverse events.

### Calculation of Savings

Postal codes were used to calculate travel distances, which were approximated based on the straight-line distance between the home address and the location of the children’s hospital.

Monetary savings were calculated using the 2022 mileage reimbursement rate of $0.69 CAD per km set by the Canadian Revenue Agency.^14^ Fuel savings were estimated using the average fuel consumption rate of 8.9 L per 100 km from the Canada Energy Regulator.^15^ A conversion factor of 0.2656 kg CO_2_-equivalent per km sourced from Canada Health Infoway was used to quantify carbon emissions avoided.^16^

### Calculation of Ontario Marginalization Index Scores

We calculated ON-Marg quintile scores according to the methodology outlined by Paczkowski et al (2024).^17^ We used the ON-Marg index to evaluate disparities in access to virtual care within the study population. Developed by Public Health Ontario from the Canadian Marginalization Index, the index groups area-level census indicators into four dimensions: households and dwellings; material resources; age and labor force; and racialized and newcomer populations.^18^

Households and dwellings include indicators that measure the following: the type and density of a residential accommodation, such as the percentage of rental dwellings and apartments; price of property; and structural characteristics of the family, such as the percentage of residents living alone, the percentage of residents who moved within five years, the number of residents per dwelling, and percentage of residents who are widowed or divorced. Material resources include indicators that measure the access and attainment of material needs such as income, the percentage of employed individuals, and the percentage of individuals with a high school degree. Age and labour force includes the dependency ratio (children < 15 and adults > 65 relative to the general population), proportion of seniors and children, and labor force participation. Racialized and newcomer populations includes the percentage of recent immigrants and the percentage of individuals who self-identify as belonging to a minority group.

We assigned each Ontario neighborhood a quintile score from 1 (least marginalized) to 5 (most marginalized) for each domain. Higher values indicate greater levels of marginalization, where each quintile represents one-fifth of the geographic units. A score of 5 therefore represents the most deprived 20% of regions in Ontario. We compared ON-Marg quintile scores for the above domains between patients who attended in-person and those who opted for virtual care to evaluate disparity in access to virtual care. Comparisons were based on postal code data and conducted across the four-fiscal-year study period. Additionally, we examined ON-Marg scores annually to address changes in the sociodemographic characteristics from year-to-year.

### Data Analysis

We summarized study outcomes with descriptive statistics including frequency statistics, means, and standard deviations. Unpaired *t* tests were conducted to compare outcomes between in-person and VUC visits. Mann-Whitney U tests were conducted to compare ON-Marg quintile values as quintile values are ordinal. One-way analysis of variance (ANOVA) with a post hoc Bonferroni test was used to assess differences between savings across fiscal years. Statistical significance was set at α = .05.

## RESULTS

A total of 12,900 VUC visits were completed during the study period, 99.9% of which were conducted via the Webex platform. Although data was not available for the entire study period, data collected between July 2023–April 2025 indicate 10.6% (353) of VUC visits resulted in a recommendation to seek care in-person at an ED. No serious adverse events were reported through the standardized postvisit feedback surveys during this period.

### Economic Outcomes

Across all five fiscal years (2020–2025), the pediatric VUC program generated an estimated $998,100 in cost savings (Table 1). Although the total savings varied in proportion to the number of visits per year, average per-visit savings increased steadily, peaking at $118.30 in 2024–2025 (Table 2).

### Environmental Outcomes

The program was associated with cumulative savings of 128,700 L of fuel and a reduction in emissions of 384,200 kg CO_2_ eq (Table 1). Per-visit savings increased annually, reaching 15.3 L of fuel and 45.52 kg of CO_2_ per visit in 2024–2025 (Table 2). Figure 1 illustrates both the annual total savings and the annual per-visit savings. While total savings generally reflected program volume, the increase in per-visit savings over time suggests that the average travel distance avoided per virtual visit increased during the study period.

### Social Outcomes

There were no significant differences in ON-Marg index quintile scores between patients presenting to VUC versus in-person ED visits for household and dwelling, age and labor force, or racialized and newcomer populations domains (Table 3). Summary ON-Marg scores were similar overall (2.91 for VUC vs. 2.87 for in-person visits) (Table 4).

### Travel Distance

Figure 2 depicts visit density by patient travel distance. The mean travel distance was 40.6 km, with greater per-visit fuel and CO_2_ savings observed year-over-year.

## DISCUSSION

To our knowledge, this is the first study to evaluate a pediatric VUC program using the SusQI framework. Our findings show that VUC is a cost-effective, environmentally sustainable, and accessible model of care. We found that the vast majority of cases did not result in in-person presentation, suggesting VUC can reduce the burden on healthcare systems by addressing low-acuity patient concerns. Over the five-year study period, VUC was associated with an estimated reduction of 384,200 kg CO_2_ eq and an estimated $998,100 CAD in avoided patient travel costs. For context, the total 384,200 kg CO_2_ eq is equivalent to the annual electricity consumption of about 80 households or 426,775 lbs of coal burned, according to the Greenhouse Gas Equivalencies calculator.^19^

The VUC program’s reductions represented 0.73% of total hospital emissions as given by our hospital’s 2021 annual emissions of 54,439 tonnes CO_2_.^20^ It is worth noting that this number may significantly underrepresent the actual environmental benefit, as it does not account for decreased CO_2_ emissions from travel avoided by clinicians, or the consumption of electricity and medical resources associated with in-person care. There were also clear economic benefits, with an estimated $998,100.00 in travel costs saved over the five fiscal years. Our findings are consistent with the literature. A previous cost analysis of VUC in an American setting found an associated savings of $93.657 USD ($132.80 CAD) per visit.^21^ Similarly, a study of 63 million virtual care visits in Ontario found that virtual care averted approximately 3.2 billion km of patient travel, or 545–658 million kg of CO_2_ eq.^22^

We found that VUC usage peaked between the 2021–2022 and 2022–2023 fiscal years, likely reflecting the COVID-19 pandemic. Virtual health and telehealth visits increased significantly during the COVID-19 pandemic, likely reflecting both public health measures and widespread attitudes towards in-person care during the pandemic.^25^ Although the total number of visits in 2024–2025 was below peak usage, usage increased from 2023–2024 to 2024–2025, reflecting sustained interest over time. Interestingly, total cost savings grew more rapidly than the number of patient encounters. We found that the savings per-visit increased annually, even when overall VUC usage declined from peak levels. This reflects a shift in the demographics of VUC users towards individuals living further away. It may also indirectly reflect the increasing economic burden of commuting which includes rising fuel and operating expenses.

### Deferred Emergency Department Visits

Only 10.6% (353) of VUC visits during July 2023–April 2025 resulted in an in-person ED visit. This number is lower than the 17.2% of visits resulting in ED referral found by a 2020–2021 prospective study analyzing data from VUC services at 14 ED-led pilot sites in Ontario, Canada.^9^ The higher rate of in-person presentation during that period may reflect the unique social environment of the pandemic which was not captured in our analysis of in-person visits. We hypothesize more patients presented to VUC with concerning symptoms because they were afraid to access in-person care.

It is important to note that VUC services are inherently different than the services provided at an in-person ED. Virtual urgent care clinicians are not able to provide a physical examination or access real-time laboratory testing or imaging. In addition, VUC clinicians are often unable to access previous medical records, all of which limit their ability to provide an accurate diagnosis.^23^ Similarly, the population of patients who use VUC services may also be intrinsically different than the population presenting to in-person EDs. Research has shown that the majority of VUC visits were by patients with low-acuity concerns who have access to primary care.^24^

### Equity and Access

A 2023 study by Williams et al found that telehealth was associated with widening health inequalities, with lower household income and ethnic minority associated with lower use of video health services, attributable to the cost of technology and the costs associated with missing work.^25^ Contrary to these findings, we observed no discrepancy in access to care across sociodemographic domains, as measured by the ON-Marg index, indicating that VUC remained accessible to patients with lower household income or those living in rural or underserved locations. Our results also indicated non-English speakers were equally likely to use both deliveries to access care.

Our findings also did not substantiate the findings of Hall et al who found that patients who use the ED for low-acuity problems were unable to use VUC because they did not have access to a computer or smartphone.^23^ Although we did not find a significant difference in marginalization status between patients using virtual and in-person care, we recognize that technological barriers remain a substantial challenge. Individuals accessing VUC must have WIFI and access to a device with video and microphone capability. They must also be able to install the Webex mobile application and have an email address to access the appointment link. It is possible that the small subset of patients who are most affected by technological barriers were not fully captured in our analysis, highlighting the need for more research specifically focused on accessibility.

Transportation barriers have also been shown to significantly restrict healthcare access, particularly for patients with lower incomes.^26^ Longer travel times correlate with a greater number of missed appointments and lower treatment adherence.^27^ Our analysis of travel distances complements these findings. During the 2023–2024 fiscal year, the mean travel distance was 40.61 km from the children’s hospital, suggesting VUC may decrease the burden of longer travel times. Virtual urgent care may also be more accessible because it eliminates barriers such as transportation costs and childcare, which can make attending in-person appointments prohibitive. In addition, VUC may also provide access to specialists and services not available in rural or underserved locations.

### Strengths

This study addresses an important gap in the literature by providing one of the first multi-year evaluations of pediatric VUC. To our knowledge, this is the first study that focuses on the economic and environmental impacts of VUC in pediatric emergency care.

### Clinical Implications

The findings suggest that pediatric VUC is a viable strategy to reduce unnecessary ED visits while alleviating some of the financial and logistical burdens placed on families. Cost savings extend beyond direct travel expenses to include indirect benefits such as reduced need for childcare, less time off work, and greater flexibility for families. Importantly, VUC appears to be accessible to marginalized populations, particularly those in rural areas. The use of virtual care may also provide additional benefits to both clinicians and patients, such as a reduction in unnecessary appointments, leading to decreased wait times and increased efficiency. Similarly, VUC may also increase flexibility and work-life balance. Research has shown that emergency physicians appreciated the opportunity to interact with patients outside the chaotic environment of the ED.^23^

Finally, the broader environmental benefits extend beyond quantifiable environmental savings. Virtual care signifies a transition toward more sustainable and more robust healthcare systems. The U.S. Agency for International Development (USAID) Health System Strengthening Vision 2030 outlines the need for resilient healthcare systems to adapt to “acute, time-bound events; and longer-term destabilizing dynamics.”^28^ In many regions in Canada, healthcare services have become increasingly centralized in larger urban centers, leaving smaller communities with limited local options. The reliance on urban facilities creates significant challenges for patients, even under normal conditions. Climate-related disruptions may therefore severely compromise access to care. We found that the greatest proportion of emission reduction occurred in patients living in rural areas. The reduction in travel associated with urgent care can improve overall access to care while also bolstering the continuity of care during climate-related disruptions. Virtual urgent care may also alleviate pressure on the healthcare system by diverting less urgent cases, allowing EDs to better adapt to increased demand. Our findings support the implementation of VUC as an important step towards more resilient healthcare systems.

### Research Implications

Future research should further explore the long-term cost-effectiveness of VUC. More robust environmental evaluations are also warranted, particularly research which examines the consumption of hospital resources associated with in-person care. From a social perspective, further investigation is needed into barriers such as digital literacy, technology access, and language support, as these remain potential obstacles to equitable use. In addition, the ED presents a confusing and often frightening experience for children, and it would be interesting to explore how pediatric patient experience differs between VUC and ED, as well as the attitudes and experiences of their families.

## LIMITATIONS

This study has several limitations. Although we captured clinician recommendations for in-person care and monitored adverse events through patient feedback surveys, we did not perform a formal chart review to verify clinical outcomes. As this study was designed to estimate financial and environmental savings using administrative dashboard data, it was not powered or structured to formally assess safety. Therefore, we are unable to draw definitive conclusions regarding diagnostic accuracy, missed diagnoses, or the overall safety profile of the VUC model.

Furthermore, the retrospective nature of this study meant that only data collected by the hospital virtual care dashboard could be included. While our research has shown a substantial cost savings per visit, it may not capture the full range of expenses typically associated with travelling to in-person ED visits, such as parking, travel costs, lost wages, and other indirect expenses such as food or accommodations. We also were not able to quantify patient-specific choices, such as patients who may have chosen to take alternative methods of transportation (eg, public transport or walking) or patients who might have chosen to present to their family doctors or closer nonpediatric EDs instead of our institution.

In addition, an in-person visit to a pediatric ED often incurs additional costs compared to a visit to a general ED, as parents or caregivers may need to arrange childcare for other children. The need to take time off work may compound the financial and logistical burden for families presenting to pediatric urgent care, leading to the potential for additional loss of income not associated with adult urgent care. As these variables were not included in the hospital virtual care dashboard, they unfortunately could not be included in our analysis. As our methodology used straight line distances to approximate travel distances, it is very likely it underrepresents travel time needed by car. Similarly, we estimated monetary and fuel savings using set rates. While these provide good approximations, they do not fully capture the volatility of gas prices across multiple years. Given this, and our inability to calculate indirect expenses, it is therefore likely that our results underestimate the full cost savings of VUC.

## CONCLUSION

At a time when we are facing a climate crisis, with healthcare systems seeking to reduce their environmental footprint, our findings demonstrate that pediatric VUC is a sustainable model of care. In our multi-year evaluation, VUC generated significant reductions in fuel consumption, travel time, and CO_2_ emissions, while also alleviating the burden on the ED. Importantly, VUC remained accessible across marginalized and rural populations. The real-world applications of our findings are significant and support the broader adoption of virtual care across the healthcare sector. Virtual urgent care should be considered a key component of future care models as healthcare systems build sustainability and resilience.

## Figures and Tables

**Figure 1:**
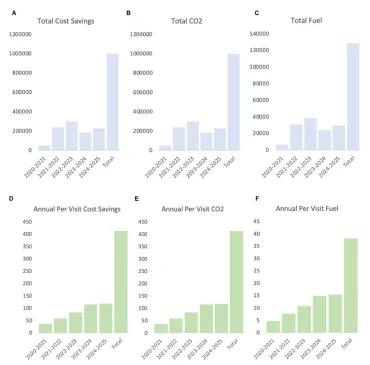
Financial and environmental savings of virtual care. Graphs A–C show annual total cost, CO_2_ eq, and fuel savings, while graphs D–F show corresponding annual per-visit savings. Total savings were driven by both the number of virtual visits and the average travel distance avoided. *CO_2_*, carbon dioxide.

**Figure 2:**
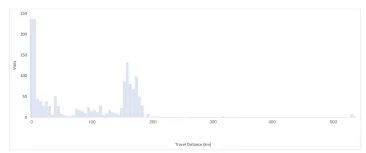
Travel distance (km) and number of visits in the 2023–2024 fiscal year. *km*, kilometre.

**Table 1:** Total savings associated with virtual urgent care during the fiscal years of 2020–2025.

Total Savings	
Number of Visits	12,900
Cost Savings ($)	$998,100.00
CO_2_ (t)	384.20
Fuel (L)	128,700

*CO_2_*, carbon dioxide; *L*, litre; *t*, tonne.

**Table 2:** Annual Savings with average per visit savings. For each outcome, the total value for each fiscal year is presented, with the average saving per visit shown in parentheses. Per-visit values were calculated by dividing the yearly savings by the number of visits in that fiscal year. Outcomes include total cost savings (in $CAD), total carbon emissions avoided (kg CO_2_ equivalent), and total fuel savings (litres).

Fiscal Year	2020–2021	2021–2022	2022–2023	2023–2024	2024–2025
Number of visits	1421	4195	3703	1624	1957
Total cost savings ($)	$50,400 ($36.60)	$237,600.00 ($59.50)	$298,700 ($83.40)	$183,800 ($115.40)	$227,600 ($118.30)
Total CO_2_ (kg CO_2 eq_)	19,400 (14.11)	91,400 (22.91)	115,000 (32.1)	70,800 (44.42)	87,600 (45.52)
Total fuel savings (L)	6,500 (4.7)	30,600 (7.7)	38,500 (10.8)	23,700 (14.9)	29,400 (15.3)

*kg CO_2_ eq*, kilograms of carbon dioxide equivalent; *L*, litre.

**Table 3:** Annual Ontario Marginalization Index Scores of patients seen virtually.

Outcome	Fiscal Year 2020–2021	Fiscal Year 2021–2022	Fiscal Year 2022–2023	Fiscal Year 2023–2024	Fiscal Year 2024–2025
Household and dwellings	2.98	2.99	3.09	3.13	3.08
Material resources	2.81	2.83	2.92	2.9	2.94
Age and labor force	2.46	2.59	2.75	2.95	2.94
Racialized and newcomer populations	3.14	3.08	3.04	2.87	2.75
Summary score	2.85	2.87	2.95	2.96	2.93

**Table 4:** Average Ontario Marginalization Index Scores of patients seen virtually versus in person across all years.

Outcome	In-Person Mean (SD)	Virtual Mean (SD)	
Household and dwellings	3.10 (0.38)	3.05 (0.07)	(*P* = .70)
Material resources	2.73 (0.52)	2.88 (0.06)	(*P* = .57)
Age and labor force	2.45 (1.00)	2.73 (0.22)	(*P* = .90)
Racialized and newcomer populations	3.22 (0.51)	2.97 (1.15)	(*P* = .49)
Summary score	2.87	2.91	

*SD*, standard deviation.
